# Orthorhombic crystal structure and oxygen deficient cluster distribution model for YBa_2_Cu_3−*x*_Al_*x*_O_6+*δ*_ superconductor

**DOI:** 10.1038/s41598-020-64535-x

**Published:** 2020-05-08

**Authors:** P. Manju, Neeraj K. Rajak, Andrews P. Alex, Vinayak B. Kamble, D. Jaiswal-Nagar

**Affiliations:** School of Physics, IISER Thiruvananthapuram, Maruthumala P.O., Vithura, Thiruvananthapuram, 695551 Kerala India

**Keywords:** Condensed-matter physics, Superconducting properties and materials

## Abstract

Single crystal x-ray diffraction measurements on both as-grown as well as oxygenated single crystals of an aluminium doped high temperature superconductor YBa_2_Cu_3−*x*_Al_*x*_O_6+*δ*_ revealed the crystal structure to be orthorhombic with space group Pmmm, in contrast to, tetragonal crystal structures corresponding to space group P4/mmm, previously reported for as-grown YBa_2_Cu_3−*x*_Al_*x*_O_6+*δ*_, and conflicting structures on oxygenated YBa_2_Cu_3−*x*_Al_*x*_O_6+*δ*_. The orthorhombic crystal structure was confirmed by powder x-ray diffraction that showed the presence of two peaks corresponding to (020) and (200) reflections associated with orthorhombic structures of space group Pmmm, instead of a single (200) reflection corresponding to tetragonal crystal structures with space group P4/mmm. All the as-grown crystals were found to be superconducting. An oxygen-vacancy cluster distribution model is proposed to explain the differences in the obtained magnetisation hysteresis loop and the broad superconducting transition temperature. The model proposes the existence of two oxygen deficient clusters of (Al-..-Cu-O-Cu)_*n*_ and (Cu-O-Cu-..)_*n*_ juxtaposed with each other whose number and size vary as the as-grown single crystals of YBa_2_Cu_3−*x*_Al_*x*_O_6+*δ*_ are subjected to oxygenation. X-ray photoelectron spectroscopy measurements showed the existence of two distinct peaks in each of the spectrum of O, Cu, Y and Ba in YBa_2_Cu_3−*x*_Al_*x*_O_6+*δ*_ crystals corresponding to the two different types of clusters. The relative intensities of each XPS peak was found to decrease in the oxygenated crystals as compared to the as-grown ones confirming the change in the number and size of clusters in the as-grown crystals after oxygenation.

## Introduction

The high temperature superconductor, YBa_2_Cu_3_O_6+*δ*_ (YBCO), has been investigated thoroughly both from a fundamental^1–5^, as well as applied point of view^6–14^. In an effort to increase the critical current density J_*c*_ of YBCO, a lot of research has gone to understand the effect of substitution of impurities of various kinds on the geometry of the main lattice. For example, while studying the effect of substitution of a cation M at the Cu site in YBa_2_Cu_3−*x*_M_*x*_O_6+*δ*_ on the superconducting properties, it was found that doping of divalent ions like Zn^2+^ and Ni^2+^ is quite detrimental to superconductivity since these ions substitute on the Cu(2) “plane” lattice site and modify the electronic structure, thereby, resulting in a strong suppression of T_*c*_ even at low doping levels^7–9^. In this regards, it is known that doping by cations M of the kind Fe^3+^, Co^3+^ and Al^3+^ are not detrimental to superconductivity at low levels of doping even though Fe^3+^ and Co^3+^ are magnetic ions, since these cations substitute predominantly on the Cu(1) “chain” lattice site. The substitution merely results in an orthorhombic to tetragonal crystal structure transformation as a function of increased cation doping^6,11–14^. By changing the preparation conditions of the as-grown crystals of aluminium substituted YBCO, YBa_2_Cu_3−*x*_Al_*x*_O_6+*δ*_ (Al-YBCO), Brecht *et al*.^12,13^, found a tetragonal crystal structure with space group P4/mmm, for all the oxidised crystals for x ≥ 0.06. A tetragonal crystal structure with space group P4/mmm was also reported by Siegrist *et al*.^6^, on measurement done on an as-grown crystal YBa_2_Cu_2.78_Al_0.22_O_6.4_ and Jiang *et al*.^11^, on an oxygenated crystal YBa_2_Cu_2.86_Al_0.14_O_7_.

To our knowledge, the effect of such structures on the superconducting properties of Al-YBCO has not been studied in detail previously. In this paper, we describe a detailed investigation of the crystal structure and superconducting properties of both as-grown as well as oxygenated single crystals of YBa_2_Cu_3−*x*_Al_*x*_O_6+*δ*_, where the Al doping was in the range of 0.09–0.16. The obtained crystal structure for both as-grown and oxygenated single crystals was found to be orthorhombic with space group Pmmm, in contrast to, tetragonal crystal structures with space group P4/mmm, in previous works^6,11–13^. The lattice constants “a” and “b” were, however, found to have quite close values. All the as-grown crystals were also found to be superconducting with the superconducting transition temperature T_*c*_ ranging between 58 K to 84 K. The magnetisation hysteresis loop widths of all the as-grown crystals were found to have a low value, irrespective of the value of the oxygen doping *δ*. However, all the oxygenated crystals were found to have a very large value of the magnetisation hysteresis loop width. In order to explain the differences of the magnetisation hystersis loop widths in the as-grown single crystals *vis a vis* the oxygenated crystals, as well as the broad superconducting transition temperatures in Al-YBCO crystals, we propose an oxygen deficient cluster distribution model. The said clusters are of two types, namely, (Cu-O-Al-..-Cu)_*n*_ and (Cu-..-Cu-O)_*n*_, each lying within the a-b plane of Al-YBCO, where the dots in (Cu-O-Al-..-Cu)_*n*_ and (Cu-..-Cu-O)_*n*_ represent the vacancy of an oxygen atom. The existence of such clusters was evidenced in spectra obtained using X-ray photoelectron spectroscopy where each spectrum of Y, Ba, Cu and O displayed a two peak character. These peaks are assumed to arise, one each, from the (Cu-O-Al-..-Al)_*n*_ clusters and (Cu-..-Cu-O)_*n*_ clusters. The large intensity ratio of the peaks corresponding to (Cu-..-Cu-O)_*n*_ clusters and the (Cu-O-Al-..-Al)_*n*_ clusters in the as-grown crystals was found to decrease in the oxygenated crystals suggesting a decrease in the number of both (Cu-O-Al-..-Al)_*n*_ and (Cu-..-Cu-O)_*n*_ clusters after oxygenation.

## Experimental Details

### Crystal growth

The technique of choice for the growth of single crystals of YBa_2_Cu_3−*x*_Al_*x*_O_6+*δ*_ is self-flux^6,7,12–25^. Accordingly, high purity Y_2_O_3_ (99.999%), CuO (99.999%) and BaCO_3_ (99.999%) powders, obtained from Merck-Aldrich, were mixed in the ratio Y:Ba:Cu = 1:18.8:46.07, equivalent to 10 wt% YBCO and 90 wt% BaO-CuO eutectic mixture (BaO:CuO = 28:72 in moles), (YBCO: flux = 1.6: 98.4 mol ratio) and put in alumina crucibles to ensure Al substitution due to crucible corrosion from the melt^6,7,12–25^. The powders were, then, heated to 1025 °C (5 °C below the peritectic melting point 1030 °C)^24^, in order to melt it and held at this temperature for ~20 hours. We employed a step cooling to minimize the multinucleation problem in order to obtain large sized single crystals^26^. In the first step, the temperature was lowered to 1005 °C at 10 °C/h and held for 0.5 hours. Crystal growth starts in the second step, where the temperature was lowered to 950 °C at a rate of 0.4 °C/h. The flux was decanted at 950 °C after which the furnace was switched off and the crystals were furnace cooled to room temperature. We employed a vertical temperature gradient^21^, for the crystal growth and used two different values of the gradient to study the differences in the properties of the grown crystals arising due to differences in the values of the employed vertical temperature gradient. The higher gradient had a value of −0.31 to −0.23 °C/mm along the height (48 mm) of crucible while the lower gradient was of the value −0.19 to −0.13 °C/mm. Crystals grown using the higher temperature gradient were labeled as V_*aH*_, V_*bH*_ and V_*cH*_ (VH batch) and those grown in lower gradient as V_*aL*_, V_*dL*_ (VL batch). The obtained as-grown crystals were oxygenated by first annealing them at 523 °C in an annealing furnace, in flowing ultra high pure (UHP) oxygen^27^. The annealed crystals, were then, taken out from the furnace and transferred to a chamber at room temperature in flowing inert gas. Surface homogenisation was done by sealing the annealed crystals in a quartz tube and annealing again at 523 °C for 20 days^24,25^.

### Measurement techniques

The grown crystals were checked for their quality, structure and magnetic properties using energy dispersive X-ray spectroscopy (EDX), inductively coupled plasma mass spectrometry (ICP-MS), single crystal X-ray diffraction (SCXRD), powder X-ray diffraction (PXRD), X-ray photoelectron spectroscopy (XPS) and vibrating sample magnetometry. EDX was measured using Nova’s electron microscope (Model: Nova NANOSEM 450) fitted with an EDS probe. ICP-MS was done at Mikroanalytisches Labor Pascher, Remagen, Germany. Pictures of the crystals were taken using an optical camera. PXRD measurements were done in a Bragg-Brentano geometry using a PANalytical Empyrean powder x-ray diffractometer, with Cu-K_*α*1_ and Cu-K_*α*2_ radiations of wavelength 1.540 Å and 1.544 Å respectively, having an intensity ratio I_2_/I_1_ of 0.5. The 2*θ* range of measurements was 5–90 degree at a step size of 0.016 degree. Magnetisation measurements were done on a vibrating sample magnetometer (VSM) connected in a physical property measurement system (PPMS) from Quantum Design (Model Evercool II).

SCXRD measurements were performed on a Bruker Kappa APEXII CCD Diffractometer, using graphite monochromated Mo-K_*α*_ radiation, having a wavelength of 0.71073 Å. The data were reduced to structure factors in the usual fashion, corrected for absorption, transformed, and averaged in the necessary symmetry. Atomic positions were located by direct methods using SHELXT programme^28^. The structure was then refined by full-matrix least-squares techniques, using the program package SHELXL^29^. Metal-atom sites were found to be fully occupied within two standard deviation units.

High resolution XPS experiments were done using a ESCA Plus spectrometer (Omicron Nanotechnology Ltd. Germany) equipment with Mg-K_*α*_ radiation (1253.6 eV). The instrument is equipped with an auto-charge neutraliser. In order to ensure that the surfaces exposed to the X-rays are clean, both the as-grown crystal as well as the oxygenated crystal were cleaned in a vacuum of 10^−10^ mbar at room temperature.

## Results and Discussion

Platelet like shiny, free standing black crystals with mirror finish surfaces, were obtained. Images of few of the extracted crystals are shown in column 2 of Table 1, while the value of the temperature gradient to which they were subjected to, in column 3. Wolf *et al*.^23^, found a constant growth front of 4 *μ*m/h along the c-axis irrespective of conditions of growth experiment, crucibles (alumina or yttria stabilized zirconia), melt dopants, etc. We found that the crystals grown using high temperature gradient had a higher value of the growth front in the c-direction (in the range 3–5 *μ*m/h) than those grown with a lower temperature gradient (see column 4 of Table 1). It can be observed from column 5 that the value of the applied temperature gradient also has an effect on the aspect ratio (length to thickness) of the grown crystals: the aspect ratio increases with decrease in temperature gradient (see Table 1) suggesting that a higher temperature gradient enhances the transport of solute particles to the growth front causing high growth rate which result in thicker crystals^23^.

**Table 1:** Extracted crystals with labels, pictures, magnitude of vertical temperature gradient, growth rate, aspect ratio and superconducting transition temperature *T*_*c*_ of the as-grown crystals.

Label	Crystal	Temperature gradient (°C/mm)	Growth rate (*μ*m/h)	Aspect ratio	*T*_*c*_ (as-grown)
V_*cH*_		−0.31 to −0.23	3–5	2.22	82.2 K
V_*aH*_		−0.31 to −0.23	3–5	4.45	84.5 K
V_*bH*_		−0.31 to −0.23	3–5	4.17	81.2 K
V_*aL*_		−0.19 to −0.13	1–2	12.91	58.3 K
V_*dL*_		−0.19 to −0.13	1–2	11.25	58 K

To quantify the amount of aluminium content in the as-grown crystals, we subjected them to EDX, ICP-MS and SCXRD measurements. Table 2 summarises the data obtained from the above mentioned techniques. Aluminium was found to be consistently in the range of 0.09 to 0.16 per formula unit irrespective of the value of the applied temperature gradient, suggesting that the melt penetration resistance of the crucible material to the chemically reactive melt is more important than the value of the applied temperature gradient for the generation of point defects in Al-YBCO.

**Table 2:** Quantification of Al content in as-grown single crystals of V_*bH*_ and V_*aL*_ using SCRD, EDX and ICP-MS analysis.

Crystal	V_*bH*_	V_*aL*_
Temperature gradient **(**°C/mm)	−0.31 to −0.23	−0.19 to −0.13
SCRD	YBa_2_Cu_2.90_Al_0.10_O_6.88_	YBa_2_Cu_2.89_Al_0.11_O_6.48_
EDX	YBa_2_Cu_2.83_Al_0.11_O_6+*δ*_	YBa_2_Cu_3.19_Al_0.10_O_6+*δ*_
ICP-MS	YBa_2_Cu_3.04_Al_0.10_O_6+*δ*_	YBa_2_Cu_3.05_Al_0.09_O_6+*δ*_

### Orthorhombic crystal structure

Single crystal X-ray diffraction measurements done on selected as-grown as well as oxygenated crystals from various batches, revealed the structure to be orthorhombic with space group Pmmm. This structure is in contrast to previously reported structures on Al-YBCO where the as-grown crystals were found to be tetragonal (having space group P4/mmm) with similar values of Al doping (x ~ 0.11) and oxygen stiochiometry (*δ* varying between 0.71 and 1)^11,13^. It is also to be noted that Brecht *et al*.^13^, found the crystal symmetry to be tetragonal for all oxygenated crystals where the Al doping ranged from x = 0.06 to x = 0.22 with the final residual ratio R_*f*_ ranging from 0.026 to 0.045. Similarly, Jiang *et al*.^11^, found the crystal structure of an oxygenated Al-YBCO crystal with x = 0.14 and *δ* = 1 to be tetragonal. On the other hand, Siegrist *et al*.^6^, found the symmetry of an as-grown crystal with an Al content of 0.22 to be tetragonal with a R_*f*_ value of 0.046. However, the symmetry of an oxygenated crystal with an Al doping of x = 0.11 was found to be orthorhombic with space group Pmmm but with a much higher R_*f*_ of 0.076. Finally, powder X-ray diffraction measurements on oxygenated Al-YBCO bulk revealed the lattice parameters of an orthorhombic structure^30^. From the above discussion, it is clear that while the as-grown Al-YBCO structures have been clearly reported to be tetragonal, there are conflicting reports on the crystal structure of the oxygenated Al-YBCO.

Table 3 describes the details of the crystal structure obtained on an as-grown tiny crystal (0.1 × 0.08 × 0.08 mm^3^) from the V_*bH*_ batch. The obtained lattice parameters are: a = 0.38470(8) nm, b = 0.38653(8) nm and c = 1.1698(2) nm with an excellent final residual value, R_*f*_ = 0.0145. As can be seen, our R_*f*_ values are much less than any previous reports^6,11,13^, indicating that the crystal symmetry of an as-grown Al-YBCO superconductor may be orthorhombic with space group Pmmm. From the Table 3, it can be found that there are 5 inequivalent oxygen sites: O_1_, O_2_, O_3_, O_4_ and O_5_. The first four oxygen sites O_1_, O_2_, O_3_ and O_4_ have been labeled in the usual way^31,32^, where O_1_ denotes the chain site, O_2_ and O_3_ label the planar oxygen sites and O_4_ labels the apical oxygen. The extra oxygen which shows occupancy at the a-site ((a,0,0) position) has been labeled as O_5_ oxygen.

**Table 3:** Crystallographic data on an as-grown YBa_2_Cu_2.89_Al_0.11_O_6.72_ crystal with an orthorhombic cell having lattice parameters *a* = 0.38470(8) nm, *b* = 0.38653(8) nm and *c* = 1.1698(2) nm.

Atom	x/a	y/b	z/c	Occupancy	U_11_	U_22_	U_33_
O_1_	0.0000	−0.5000	0.0000	0.6994	0.009(3)	0.007(3)	0.005(3)
O_2_	0.5000	−1.000	0.3772(7)	2	0.005(2)	0.007(2)	0.009(2)
O_3_	0.0000	−0.500	0.3796(7)	2	0.007(2)	0.004(2)	0.008(2)
O_4_	0.0000	0.0000	0.1565(3)	2	0.019(2)	0.018(2)	0.013(2)
O_5_	0.5000	0.0000	0.0000	0.0189	0.130(60)	0.0310(60)	0.130(60)
Cu_1_	0.0000	0.0000	0.0000	0.8877	0.013(1)	0.009(1)	0.004(1)
Al_1_	0.0000	0.0000	0.0000	0.1123	0.013(1)	0.009(1)	0.004(1)
Cu_2_	0.0000	−1.000	0.3571(1)	2	0.005(1)	0.004(1)	0.007(1)
Y_1_	0.5000	−0.5000	0.5000	1	0.006(1)	0.006(1)	0.004(1)
Ba_1_	0.5000	−0.5000	0.1870(1)	2	0.012(1)	0.007(1)	0.008(1)

Space group is *Pmmm* (No. 47); Z = 1, U_*ii*_ are the mean-square displacements; 2980 total reflections measured. R_*f*_ = 0.0145.

From the results of Table 3, it is also clear that Al substitutes the Cu(1) site exclusively, in agreement to the previous reports^6,11,13^. Occupancy of an oxygen atom at the *a* position (O_5_ site occupancy) results in a tendency towards equalisation of the lattice constants *a* and *b* due to the cross-linking of the Cu-O bonds in the *a* direction. Consequently, Al^3+^ atoms that replace the Cu(1) atoms should find themselves in an octahedral environment, the favoured co-ordination for the Al^3+^ atoms^6,13^. It is to be noted that the occupancy of O_5_ atoms at the a-site is rather low at 0.0189. A refinement with no oxygen at the a-site resulted in a higher R_*f*_ value at 0.0147. No oxygen at the a-site would also imply that Al^3+^ ions would be in a much less preferred square-planar co-ordination with four oxygen neighbours, two chain oxygens (O(1) oxygens) and two apical oxygens (O(4) oxygens). So, it seems energetically favourable for an oxygen atom to occupy an a-site and Al^3+^ ions be in an octahedral environment, consequently. Finally, we provide the details of the crystal structure of an oxygenated crystal from the V_*cH*_ batch in Table 4. It was found that all the oxygenated crystals crystallize in the orthorhombic symmetry with space group Pmmm, exactly similar to the symmetry of the as-grown crystals. Additionally, the oxygen occupancy of the O_5_ site was found to be much higher at ~0.4 in the oxygenated crystals. This would result in a greater cross-linkage of the Cu-O bond in the basal plane, thereby, resulting in a greater equalisation of the “a” and “b” lattice constants, as observed.

**Table 4:** Crystallographic data of an oxygenated YBa_2_Cu_2.84_Al_0.16_O_6.72_ crystal having orthorhombic cell with space group Pmmm and lattice parameters *a* = 0.38602(6) nm, *b* = 0.38672(6) nm and *c* = 1.17104(17) nm; 4231 total reflections measured. R_*f*_ = 0.0229.

Atom	x/a	y/b	z/c	Occupancy	U_11_	U_22_	U_33_
O_1_	0.0000	0.5000	0.0000	0.3254	0.011(5)	0.006(5)	0.003(5)
O_2_	0.5000	0.000	0.3783(5)	2	0.005(2)	0.011(2)	0.007(3)
O_3_	0.0000	0.500	0.3778(5)	2	0.010(2)	0.006(2)	0.007(3)
O_4_	0.0000	0.0000	0.1561(5)	2	0.024(3)	0.025(3)	0.007(3)
O_5_	0.5000	0.0000	0.0000	0.3950	0.005(5)	0.001(5)	0.002(5)
Cu_1_	0.0000	0.0000	0.0000	0.8365	0.013(1)	0.009(1)	0.004(1)
Al_1_	0.0000	0.0000	0.0000	0.1635	0.010(1)	0.012(1)	0.001(1)
Cu_2_	0.0000	0.000	0.3567(1)	2	0.006(1)	0.007(1)	0.006(1)
Y_1_	0.5000	0.5000	0.5000	1	0.007(1)	0.009(1)	0.003(1)
Ba_1_	0.5000	0.5000	0.1863(1)	2	0.011(1)	0.012(1)	0.007(1)

By systematically measuring PXRD on Co doped YBCO (YBa_2_Cu_3−*x*_Co_*x*_O_6+*δ*_) powders^14^, as a function of the doping concentration x, it was found that the PXRD lines corresponding to (200) and (020) planes were well-separated for x ≤ 0.075 and the structures corresponding to these concentrations were orthorhombic. For x ≥ 0.075, the lines merged and the corresponding structures were tetragonal. The spontaneous strain, e = 2 * (b − a)/(b + a), arising due to structural transition from tetragonal to orthorhombic in undoped parent YBCO, was also found to reduce in magnitude at the cross-over concentration. A similar observation was also made in Al doped YBCO (YBa_2_Cu_3−*x*_Al_*x*_O_6+*δ*_)^11,18^, and Fe doped YBCO (YBa_2_Cu_3−*x*_Fe_*x*_O_6+*δ*_)^9^, where the cross-over concentration was found at x ~ 0.12 and 0.09 respectively. So, our crystals of Al-YBCO seem to be near the critical concentration of orthorhombic to tetragonal structural transition.

**Figure 1:**
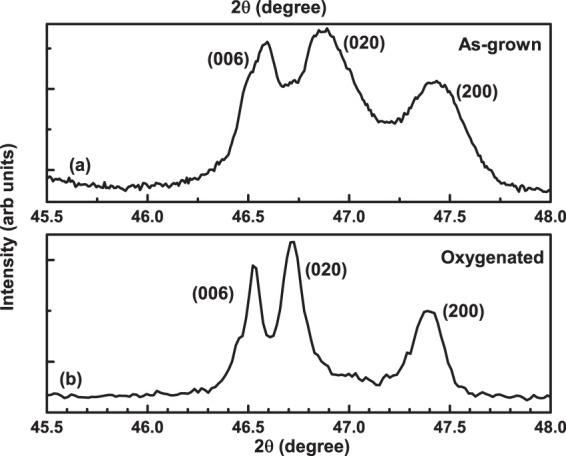
Powder X-ray diffraction peaks (006), (020) and (200) of YBa_2_Cu_3−*x*_Al_*x*_O_6+*δ*_ as a function of 2*θ* in the range 45.5° < 2*θ* < 48° for (**a**) crushed as-grown single crystals and (**b**) an oxygenated crushed single crystal.

By measuring temperature dependent PXRD on an Iron (Fe) chalcogenide superconductor Fe _1.13_Te^33^, it was found that when the sample had a tetragonal crystal structure with space group P4/mmm, the PXRD pattern exhibited a single (200)_*T*_ reflection at 2*θ* ~ 47.6° above a temperature of 55 K. However, as soon as the temperature was reduced below 50 K, the (200)_*T*_ peak split into two (200)_*O*_ and (020)_*O*_ reflections, signaling a tetragonal to orthorhombic structural transition with space group Pmmm. Similarly, from synchrotron x-ray powder diffraction measurements, Ali *et al*.^34^, confirmed a structural phase transition between a low temperature orthorhombic (Pmmm) phase and a high temperature tetragonal (P4/mm) phase in La_0.63_(Ti_0.92_,Nb_0.08_)O_3_ by the presence of an additional peak denoting (020) reflection corresponding to an orthorhombic phase apart from the (200) peak which was only present in the tetragonal phase. So, in order to confirm the orthorhombic crystal structure of our as-grown as well as oxygenated Al-YBCO, we performed PXRD on few single crystals after crushing them. Figure 1(a) shows the PXRD diffractogram for the as-grown crystals while Fig. 1(b) shows the powder diffractogram of an oxygenated crushed single crystal, measured in the relevant 2*θ* range of 45.5° < 2*θ* < 48° at a step size of 0.0176. It can be clearly seen from both the figures that the (006), (020) and (200) peaks are all well separated confirming the orthorhombic nature of the crystal structure for both as-grown as well as oxygenated crystals. It is also to be noted that the PXRD peaks in Fig. 1(a) are broader compared to the ones in Fig. 1(b) since the former is a powder X-ray diffractogram obtained on a collection of as-grown single crystals from the same batch while the powder diffractogram obtained in Fig. 1(b) is taken on a single oxygenated crystal that was crushed. The sharp peaks of Fig. 1(b) point to the excellent quality of the grown single crystals. Hence, it is clear that if the crystal structure is orthorhombic, then the powder X-ray diffractogram would show the existence of two peaks representing (200) and (020) reflections, irrespective of the PXRD being done on a single crushed crystal or a collection of them, as observed.

### Oxygen vacancy cluster distribution model

**Figure 2:**
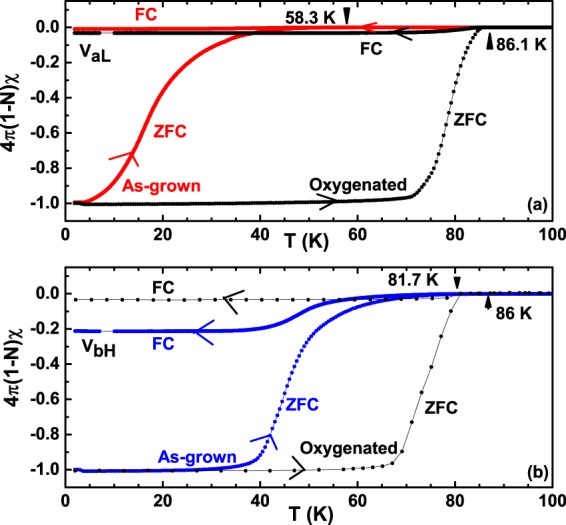
Effect of oxygenation on the superconducting transition temperature T_*c*_ of as-grown single crystal grown in (**a**) lower temperature gradient and (**b**) higher temperature gradient.

In order to probe the superconducting properties of the grown Al-YBCO single crystals, we have measured the dc magnetic susceptibility and magnetisation hysteresis loops (MHL’s) of over 20 crystals grown in different batches. It was found that all the crystals grown using the higher vertical temperature gradient show a T_*c*_ in the range 82–84 K while crystals grown using the lower temperature gradient have T_*c*_ ~ 58 K. Red curve in Fig. 2(a) shows the *dc* magnetic susceptibility of an as-grown crystal V_*aL*_ obtained using a VSM. For the measurements, the crystal was first cooled in a zero field to the lowest temperature of 1.9 K and then a field of 1 mT was applied for H || c. Data was collected while warming up the sample (zero-field cooled (ZFC) warm-up) as shown by red arrow in Fig. 2(a). Field cooling data from the normal state in the same applied field of 1 mT (Field-Cooled (FC)) are also shown and marked as FC in Fig. 2(a). It can be seen that the as-grown crystal V_*aL*_ is superconducting with a T_*c*_ (onset) ~ 58.3 K and a very broad superconducting transition width (ΔT_*c*_) ~ 40 K (10–90%). Similarly, blue curve in Fig. 2(b) plots the ZFC and FC magnetisation of an as-grown crystal V_*bH*_, grown using the higher temperature gradient. In this case, the T_*c*_ (onset) is higher at ~81.7 K but with a similar width in the transition temperature (~40 K). So, a higher T_*c*_ exhibited by the as-grown crystals in the VH batch implies a higher oxygen content in these crystals that results from a poorer insulation in the higher temperature gradient furnace.

The ZFC curves of all the as-grown crystals grown either in low-gradient or high-gradient have large superconducting transition width, in accordance with other reports^15,20–22^, suggesting that, the oxygen is distributed very inhomogeneously in the entire crystal^25^. However, the width of the transition temperature is similar (~40 K) suggesting that the reason for the broad width is independent of the gradient used in growing single crystals of Al-YBCO. In order to reduce the superconducting transition width as well as improve the oxygen content, both the crystals V_*aL*_ and V_*bH*_ were oxygenated according to the details in section 2.1. Black curves in Fig. 2 show the resultant dc magnetic susceptibility. As is immediately evident, the ΔT_*c*_ has reduced substantially to ~9 K and T_*c*_ increased to 86 K. The annealing conditions were for an oxygen content *δ* of 0.92 corresponding to highest T_*c*_ (92 K) obtained from the Lindemer’s diagram^27^, that was made for the parent YBCO. So, a lower value of T_*c*_ ~ 86 K on the oxygenated crystals V_*aL*_ and V_*bH*_ suggests that the obtained value of *δ* in these oxygenated crystals is lower. From SCXRD, this value was obtained as 0.72. A similar observation was also made by Brecht *et al*.^13^, where a careful oxygenation of their Al-YBCO crystals also resulted in a lower value of *δ* than expected for the parent YBCO. It was argued by Brecht *et al*.^13^, that Al^3+^ replacement of Cu(1) ions resulting in an octahedral environment for Al^3+^, also results in a five-fold co-ordination for Cu(1) which is not favourable for Cu(1). So, the excess oxygen ions should leave the lattice reducing the overall oxygen content of Al-YBCO, as observed.

**Figure 3:**
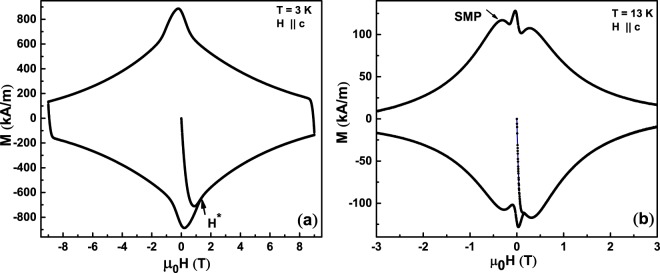
Magnetic hysteresis loops measured on an as-grown crystal V_*bH*_, with the applied field *μ*_0_H || *c* at (**a**) 3 K & (**b**) 13 K.

Figure 3(a) shows a five quadrant magnetisation hysteresis loop (MHL) of an as-grown crystal V_*bH*_ of Al-YBCO using a VSM in a PPMS at T = 3 K for *μ*_0_H ~ c. The data were collected while ramping the field at a rate of 0.1 T/min. The field of full penetration is ~1.4 T and is marked by arrows as H* in Fig. 3(a). Figure 3(b) shows the M-H loop for the same crystal at an elevated temperature of 13 K. Very interestingly, one can now notice the presence of a second magnetization peak (SMP) anomaly, at a field of ~0.27 T. The SMP anomaly was observed in all the as-grown crystals above 8 K, irrespective of the gradient in which they were grown. It was found that the onset as well as peak field of the SMP anomaly is highly field ramp direction dependent^35^, suggesting the possibility of the co-existence of two different kinds of clusters having slightly different pinning characteristics.

**Figure 4:**
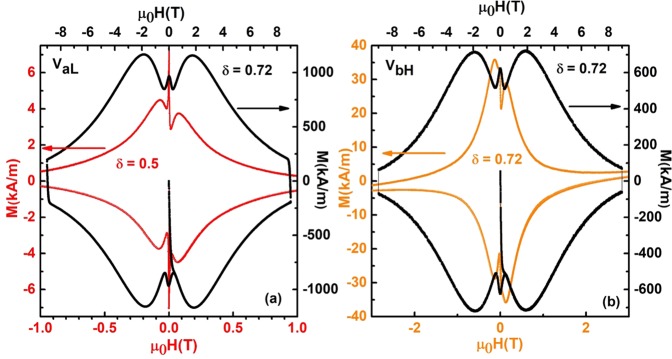
(**a**) Red and (**b**) Orange curves denotes MHL in as-grown crystals V_*aL*_ and V_*bH*_ respectively at T = 25 K. Right and top labels correspond to data on V_*aL*_ and V_*bH*_ crystals after oxygenation shown as black curves.

In order to explore this idea further and also understand the effect of oxygenation on the MHL of the as-grown Al-YBCO crystals, we measured MHL’s on many crystals two of which are described in this section. The crystals were chosen one each from low gradient as well as high gradient, viz. crystal V_*aL*_ and V_*bH*_ respectively. Coloured lines in Fig. 4 show the MHL recorded on as-grown V_*aL*_ (red curve) and V_*bH*_ (orange curve) crystals while black lines correspond to the MHL on the corresponding as-grown crystals that were subsequently oxygenated.

It can be immediately noted that, the width of the hysteresis loop has increased considerably. Since the width of MHL is a measure of J_*c*_^36^, the increase in width of MHL implies that the current carrying capacity of the crystals V_*aL*_ and V_*bH*_ has increased quite a lot after oxygenation. This seems counter-intuitive since J_*c*_ $\propto$ $\overrightarrow{\nabla }$ × $\overrightarrow{B}$ and an increased oxygenation, should supposedly, reduce the gradient in magnetic induction by filling up the oxygen deficient sites. Additionally, the oxygen content for both the as-grown as well as the oxygenated crystal V_*bH*_ is the same (*δ* ~ 0.72), however their magnetisation hysteresis loop widths are entirely different (see Fig. 4(b)). This observation suggests that oxygen distribution should be different for these crystals having the same values of oxygen deficiency (*δ* = 0.72)^37^.

**Figure 5:**
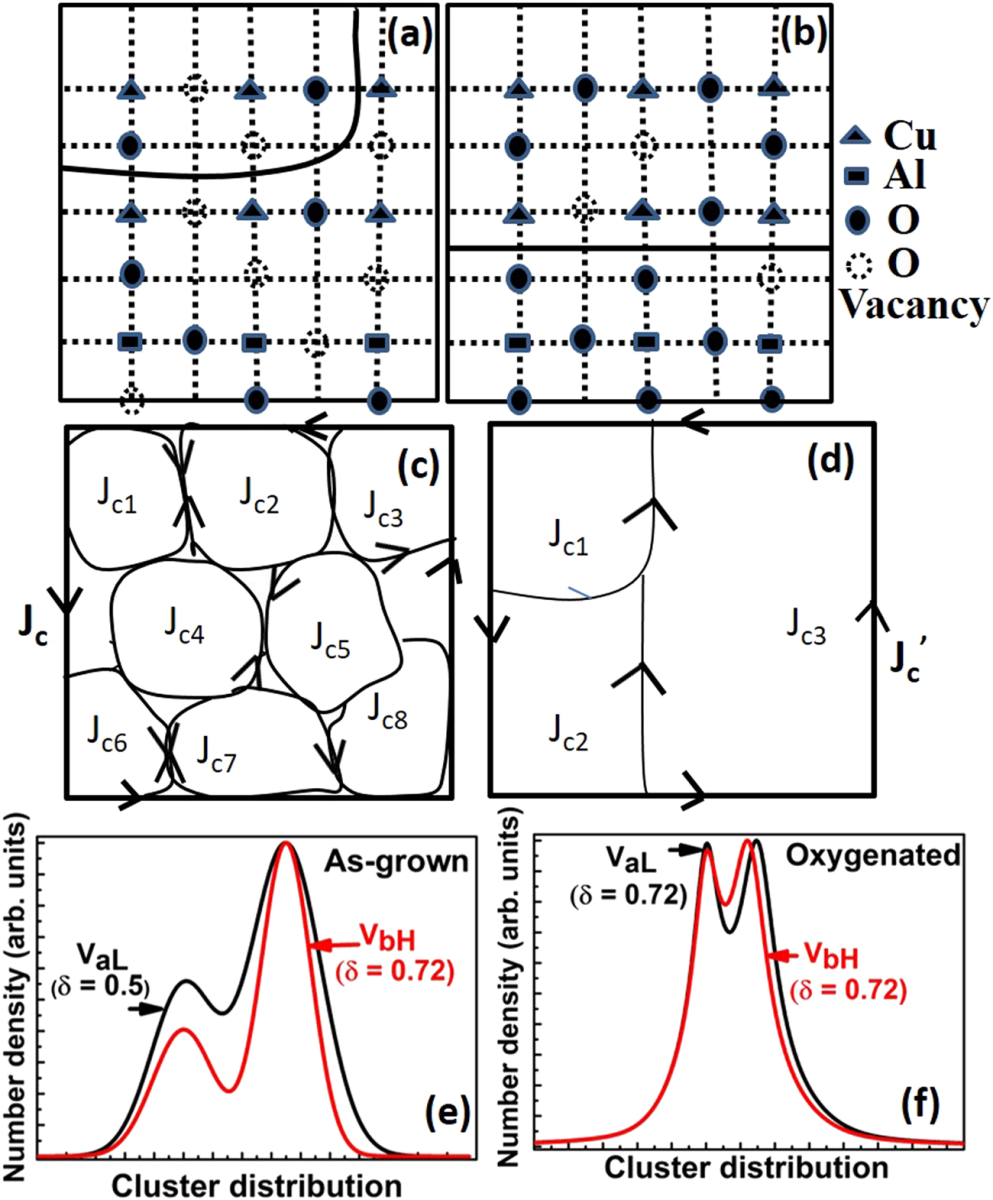
Schematic of two kinds of clusters in the a-b plane of (**a**) as-grown crystals and (**b**) oxygenated crystals of YBa_2_Cu_3−*x*_Al_*x*_O_6+*δ*_. Top clusters shown in (**a** and **b**) represent (Cu-..Cu-O)_*n*_ clusters where the.. represents an oxygen vacancy shown as open dotted circles, while the bottom clusters represent (Cu-O-Al-..Cu)_*n*_ clusters. Schematic of critical currents J_*ci*_ flowing through clusters in (**c**) as-grown and (**d**) oxygenated crystals. Bi-modal cluster distribution representing (Cu-O-Al-..Cu)_*n*_ and (Cu-O-Al-..Cu)_*n*_ clusters in (**e**) as-grown V_*aL*_, V_*bH*_ crystals and (**f**) after oxygenation.

To understand the above mentioned observations, we propose the formation of oxygen deficient clusters of (Al-..-Cu-O-Cu-)_*n*_, lying in juxtaposition to the oxygen deficient main lattice of (Cu-..-Cu-O)_*n*_, in the basal plane of Al-YBCO crystals. The dots (..) depicted in the clusters above represent the lack of oxygen at their expected site and are assumed to be statistically distributed. The existence of such clusters was also proposed in^13,30^, as well as in Co-substituted YBCO^38^. Figure 5(a,b) show a schematic of such clusters in the basal plane a-b where Al is shown to substitute Cu. While Fig. 5(a) shows clusters in an as-grown crystal, Fig. 5(b) show clusters in the oxygenated crystal. Oxygen is shown to be missing from various expected sites, since the as-grown crystals are quite deficient in oxygen. It can be seen that there are two different kinds of clusters, one containing (Al-O-Cu-..) while the other containing only (Cu-O-Cu-..). The two clusters are shown to be separated from each other from a curved line (drawn for brevity in Fig. 5(a)) and solid line in Fig. 5(b). The size and distribution of each cluster is assumed to vary from crystal to crystal. Each of such clusters have currents of magnitude *J*_*ci*_ (where i denotes the i^*th*^ cluster) running around them, such that a net total current of magnitude J_*c*_ and ${{\rm{J}}{\prime} }_{c}$ flow in as-grown and oxygenated crystal respectively, as shown in Fig. 5(c,d). Since the as-grown crystals have a large number of oxygen deficient sites, they consequently, have a large number of such oxygen deficient clusters (see Fig. 5(c)). Our proposition is that these clusters, are distributed in a bi-modal distribution, as shown in Fig. 5(e,f). Each mode of the distribution signifies the number and size of (Al-..-Cu-O-Cu-)_*n*_ cluster and (Cu-..-Cu-O)_*n*_ cluster. Since the as-grown crystals have a large size distribution of both kinds of clusters, the full width at half maximum of each of the mode is large (see Fig. 5(e)). Each of the large number of these clusters would superconduct at slightly different temperatures giving rise to a large transition width as observed in dc magnetic susceptibility of as grown crystals (refer to Fig. 2). Many of the counter-flowing currents in these clusters would cancel each other, reducing the magnitude of the total current carried by such as-grown crystals. Hence, their magnetisation hysteresis loops have very small widths, as observed in the as-grown crystals of V_*aL*_ and V_*bH*_ (see Fig. 4(a,b)). One could argue that the large ΔT_*c*_ observed in as-grown as well as oxygenated crystals could arise from differing crystallites of Al-YBCO if the crystal quality is not good, and not necessarily from clusters of the kind mentioned above. However, our excellent single crystal XRD data which gave very small final residual values of the order of 0.0145 (see details in sub-section “Orthorhombic crystal structure” above) gives us confidence that the clusters arise in one single crystal of Al-YBCO and that our single crystals are not multiply connected crystallites.

When the as-grown crystals are oxygenated, the oxygen deficient sites get filled with oxygen resulting in a decrease in the size distribution of both the (Al-..-Cu-O-Cu-)_*n*_ clusters as well as (Cu-..-Cu-O)_*n*_ clusters. This is shown schematically in Fig. 5(b) where the oxygen deficient sites, represented by open circles, have reduced to quite a few. Because of Al^3+^ substitution and a consequent loss of oxygen from the ab plane (refer discussions above), there would still be oxygen vacant sites in oxygenated Al-YBCO, albeit, with much reduced number density as compared to as-grown crystals. Hence, the earlier small sized clusters would, coalesce together to form few large sized cluster, as shown in Fig. 5(d). These large sized clusters, would then, have large magnitude of currents flowing across them resulting in the oxygenated crystals having a large value of magnetisation hysteresis loop width, as observed in Fig. 4(a,b). Consequently, the oxygenated crystals have a smaller full width at half maximum at each of their modes since both (Al-..-Cu-O-Cu-)_*n*_ cluster and (Cu-..-Cu-O)_*n*_ cluster have a narrower cluster distribution now, as shown in Fig. 5(f). The small number of clusters would, then, lead to a smaller superconducting transition width ΔT_*c*_ in the oxygenated crystals, as observed in Fig. 2.

One of the observable effect of the existence of the two types of clusters of (Al-..-Cu-O-Cu-)_*n*_ and (Cu-..-Cu-O)_*n*_ was the first ever observation of, field direction dependent SMP anomaly in as-grown crystals of Al-YBCO^35^, where the differences in fields of the onset and peak of SMP anomaly was supposed to arise due to differences in the pinning mechanism of the two kinds of clusters. If the proposed (Al-O-Cu-O..-Cu-)_*n*_ clusters are different from the (Cu-O-Cu-..)_*n*_ clusters, then this difference could be observed from core-level shifts in x-ray photoelectron spectroscopy (XPS) measurements, a very sensitive technique for probing differences in binding energies of elements according to their local environment.

**Figure 6:**
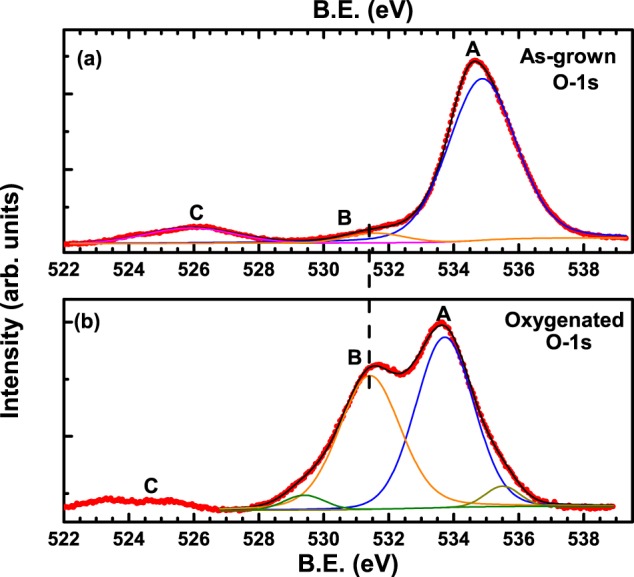
O-1s core-level spectrum of (**a**) as-grown and (**b**) oxygenated V_*bH*_ crystal. Red filled circles are the data points while the black envelope is a fit. Resolved peaks are shown by thin coloured lines.

Figure 6(a) shows the O-1s core-level spectrum of as-grown V_*bH*_ crystal, wherein, the filled red circles correspond to data points. Three distinct features marked as A, B and C can be clearly observed. The feature corresponding to C has been observed in various other non YBCO samples and are ascribed to arise due to surface contaminants. Taking a cue from this data, we cleaned the surfaces of oxygenated crystals even better than the one for as-grown crystal before taking the XPS spectra. It can be clearly seen from Fig. 6(b) that the feature C goes away after the cleaning treatment lending credence to the argument that the feature C arises due to contaminants at the surface.

XPS measurements on oxygenated single crystals^32,39,40^, thin films^41,42^, or bulk samples^43–45^, of parent YBCO have been reported by many groups. They report an O-1s main peak in the binding energy (B.E.) range of 528 eV^39,40^, to 528.5 eV^32,43,45^, with a shoulder in the range of 527.5 eV^32^, to 530.5 eV^39,40,43^, to 531.5^45^. Since oxygen is at four inequivalent sites in parent YBCO (planar O(2) and O(3) sites; chain O(1) and apical O(4) sites), in principal, one could distinguish between all the four oxygen atoms. However, since the binding energy differences between O(4) and all the other oxygen atoms are not much, only two peaks get resolved in the YBCO spectra. Maiti *et al*.^32^, ascribe the main peak at 528.5 eV to the three oxygen atoms (O(1), O(2) and O(3)) from the bulk of the crystal, while the lower binding energy peak at 527.5 eV was assigned to the apical oxygen O(4). It is to be noted that this assignment was done for YBa_2_Cu_3_O_6+*δ*_ with *δ* = 1. It was also found that with a decrease in the oxygen content *δ*, both the main peak as well as the shoulder peak shift to higher B.E. values^32^. In our Al substituted and oxygen deficient (Al-..-Cu-..-Cu-)_*n*_ cluster model, since Al^3+^ ions replace Cu(1) in the basal plane, so all the five oxygen atoms O(1), O(2), O(3), O(4) and O(5) atoms would experience two different environments; one with respect to the Cu(1) atoms and the other with respect to Al atoms. So, we expect two peaks in the XPS spectra of Al-YBCO, as observed in the form of features A and B in Fig. 6. Zhang *et al*.^45^, measured the XPS spectra of an Al-doped YBCO, YBa_2_Cu_2.9_Al_0.1_O_7.1_, in the bulk form and found a very high intensity O-1s peak at 531.5 eV with an almost disappearing tiny shoulder at 525.5 eV, suggesting that Al substitution results in a suppression of the parent YBCO O-1s peak at 528.5 eV. However, the authors did not make any assignment for the higher B.E. peak. By doing grazing incident XPS studies on defective CeO_*x*_ films, Holgado *et al*.^46^, found an additional O-1s peak at a higher B.E. that was ascribed to the oxide ions in the defective CeO_*x*_ films. So, it is probable that the higher B.E. peak in^45^, corresponds to the oxygen atoms associated with the substituted Al atoms. As proposed earlier, the as-grown crystals of Al-YBCO have a large number of (Al-..-Cu-O-Cu-)_*n*_ clusters where the position of Al as well as O is statistically distributed. These (Al-O-Cu-..-Cu-)_*n*_ clusters lie in juxtaposition to the main (Cu-..-Cu-O)_*n*_ lattice which is also oxygen deficient. So, the lower binding energy feature B in our O-1s XPS spectra is assigned to the (Cu-..-Cu-O)_*n*_ clusters while the feature A is assigned to the (Al-O-Cu-..-Cu-)_*n*_ clusters. Black line in Fig. 6(a) corresponds to a fit using XPS Peak software while the orange and blue thin lines are the resolved peaks. Since the clusters are of differing sizes and numbers in the entire Al-YBCO crystal, the B.E. of corresponding oxygen atoms should be different from the parent YBCO, as observed.

Figure 6(b) shows the O-1s core level spectrum of V_*bH*_ crystal after it was oxygenated. The difference between the as-grown spectrum and the one obtained after oxygenation is immediately apparent (c.f. Fig. 6(a,b)). The broad and low intensity peak of feature B in the as-grown crystal has now transformed to a much higher intensity peak in the oxygenated crystal. Additionally, the higher B.E. peak at 534.8 eV in the as-grown crystal has now shifted to lower binding energies. It is also to be noted that the feature B in the spectrum of the oxygenated crystal can now be resolved in two peaks- one high intensity peak at 531.4 eV and a smaller intensity peak at the lower B.E. of 529.4 eV. The higher B.E. peak of 531.4 eV is at the same B.E. as that observed in the as-grown spectra (shown by vertical dashed line). As has been proposed earlier, once the crystals are oxygenated, the large number of (Al-O-Cu-..-Cu-)_*n*_ clusters as well as (Cu-..-Cu-O)_*n*_ clusters transform to a very small number of large sized (Al-O-Cu-O-Cu-)_*n*_ and (Cu-O-Cu-O)_*n*_ clusters. Hence, the relative intensities of the peaks corresponding to feature A and B should decrease when compared to the as-grown spectrum, as observed. The lower intensity peak at 529.4 eV in the spectrum of Fig. 6(b) is ascribed to the O(4) atoms while the peak at 531.4 eV is ascribed to the remaining oxygen atoms, guided by the markings of Maiti *et al*. in^32^. In contrast, the lower intensity peak in the resolved spectrum corresponding to feature A and supposedly arising due to (Al-O-Cu-O-Cu-)_*n*_ clusters, occurs at a higher B.E. of 535.5 eV compared to the higher intensity peak at lower binding energy of 533.7 eV. So, we cannot make a similar assignment of various oxygen atoms corresponding to the feature A of Fig. 6(b) as that of feature B.

**Figure 7:**
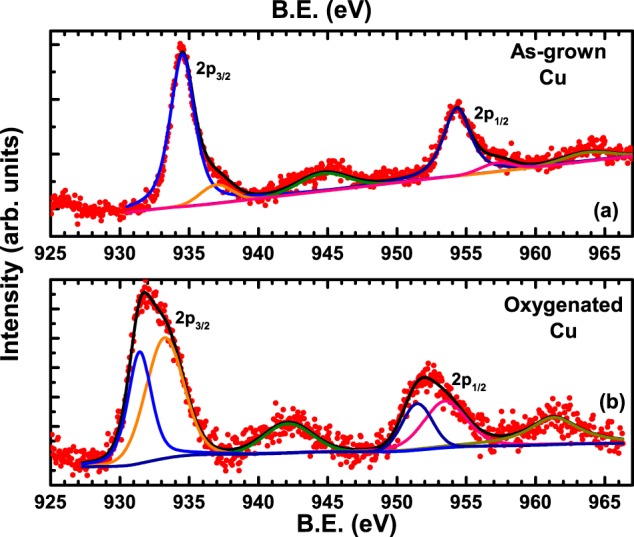
Cu-2p_3/2_ and 2p_1/2_ core level spectrum in (**a**) as-grown and (**b**) oxygenated V_*bH*_ crystal. Satellite peaks corresponding to Cu-2p_3/2_ and 2p_1/2_ core levels can be seen.

Next, we show the Cu 2p core level spectrum in Fig. 7, where Fig. 7(a) shows the spectrum of as-grown Al-YBCO crystal V_*bH*_ while Fig. 7(b) shows the spectrum of V_*bH*_ after it was oxygenated. Both the spectra are characterised by four spectral features: two high intensity peaks corresponding to 2p_3/2_ and 2p_1/2_ spin-orbit split levels and two lower intensity peaks that are the satellites of the corresponding spin-orbit levels. It is well known that in divalent cuprate compounds where copper has the electronic configuration Cu^2+^: [Ar]3d^9^, there occurs a single hole in the $3{{\rm{d}}}_{{x}^{2}-{y}^{2}}$ orbital per copper atom^47,48^. This is an energy intensive process due to the large on-site Coulomb repulsion. So, creating a hole in the Cu-2p orbital results in a lowering of Cu-3d energy levels resulting in a charge transfer from the surrounding oxygen ligand orbital to copper as a screening response to the core hole^49^. So, each spin-orbit component of Cu-2p core level line of divalent cuprates splits into a main peak with configuration c^−1^3d^10^L^−1^ and a satellite peak with configuration c^−1^3d^9^, where c^−1^ corresponds to hole in core level and L^−1^ corresponds to ligand hole in surrounding O-2p levels. Since the doping parameter *δ* is of the order of 0.72 for the as-grown as well as the oxygenated crystal, the chain Cu(1) must be in an admixture of Cu^+2^ as well as Cu^+3^ states^48^, so the main Cu-2p peaks should show satellite peaks (due to the presence of Cu^2+^ ions) for each spectral line, as observed. However, the presence of two different kinds of Cu (chain and planar) with differing oxidation states and dimensionality makes it difficult to assign a particular spectral line to a specific site.

Black curves superimposed on the red data points in each curve of Fig. 7 is a fit comprising six peaks. The peaks shown by olive green line at a B.E. of 944.7 eV and dark yellow line at a B.E. of 963.7 eV correspond to the satellite peaks of the main 2p_3/2_ and 2p_1/2_ spin-orbit lines respectively. The most intense peak in Fig. 7(a) at a B.E. of ~934.5 eV corresponds to the spin-orbit line of 2p_3/2_, while that at ~954.3 eV corresponds to that of 2p_1/2_. It is to be noted that both the spin-orbit lines of Fig. 7(a) can be resolved into two peaks: a lower intensity peak (shown in pink and orange corresponding to 2p_3/2_ and 2p_1/2_ respectively) and a higher intensity peak (shown in blue and dark-blue corresponding to 2p_3/2_ and 2p_1/2_ respectively), similar to the observation of two split peaks in oxygen spectra (c.f. Fig. 6). Both the resolved peaks of the 2p_3/2_ spin-orbit line as well as 2p_1/2_ line were found to be in the expected ratio of 2:1. As discussed above, the higher binding energy peaks should correspond to the (Al-O-Cu-..-Cu-)_*n*_ clusters while the lower binding energy peaks should correspond to the (Cu-..-Cu-O)_*n*_ clusters. According to our model, after oxygenation, the relative ratios of the peak intensities should change due to a filling up of oxygen vacant sites in each (Al-O-Cu-..-Cu-)_*n*_ cluster as well as (Cu-..-Cu-O)_*n*_ clusters. This is exactly what is observed in Fig. 7(b) where the intensity of lower intensity peaks corresponding to 2p_3/2_ and 2p_1/2_ spectral features of Fig. 7(a) (pink and orange lines) are found to increase to a magnitude equal to that of the corresponding high intensity features (blue and dark blue lines) of Fig. 7(b). The composite peaks of the spin-orbit lines 2p_3/2_ and 2p_1/2_ in our Al doped YBCO occur at ~932 eV and 952 eV respectively, while those reported in the parent YBCO occur around 933 eV for 2p_3/2_ line^32,40,43^.

**Figure 8:**
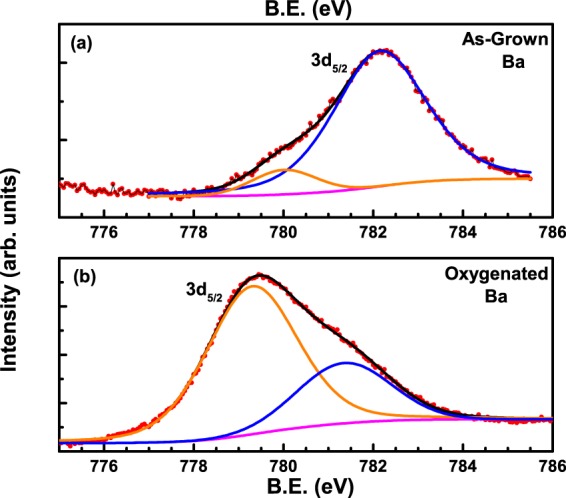
Fit to the Ba-3d core level spectrum of (**a**) as-grown and (**b**) oxygenated V_*bH*_ crystal.

Moving on to the Ba-3d spectra, Fig. 8(a) shows the 3d_5/2_ core-level spectrum for the as-grown crystal V_*bH*_. Red filled circles correspond to the data points while the superimposed black curve is a fit to the spectrum. The fit reveals two peaks, a low intensity peak (shown in orange) at ~780 eV and a high intensity peak at ~782.2 eV, similar to the observations of two peak structures in the spectra of oxygen and copper (c.f. Figs. 6 and 7). So, the proposed oxygen deficient clusters of the kind (Al-O-Cu-..-Cu-)_*n*_ that affect the oxygen XPS spectra (c.f. Fig. 6) are bound to have an affect on the Ba spectra, as observed. Similar to the assignments above, we assign the lower binding energy peak to the (Cu-..-Cu-O)_*n*_ clusters of the main lattice while the higher binding energy peak to the clusters of the kind (Al-O-..-Al-Cu-)_*n*_. The crystal structure of Al-YBCO reveals 12 oxygen atoms co-ordinating with one Ba atom- four oxygen within the Ba plane, four above it and four below. The presence of extra O(5) atoms in the a-b plane result in two extra co-ordinations in Al-YBCO crystals compared to the parent undoped YBCO.

Figure 8(b) shows the Ba-3d_5/2_ spectrum of V_*bH*_ crystal after it was oxygenated. Orange and blue lines correspond to the deconvoluted peaks at 779.3 eV and 781.4 eV respectively, arising from the fit. A Ba-3d_5/2_ peak has been reported in the range of 778 eV to 782 eV in single crystals of parent YBCO^32,40,43^, while Zhang, *et al*.^45^ report a Ba-3d_5/2_ peak at 780 eV in an Al doped powder of YBCO. The difference in the spectrum obtained on the oxygenated crystal compared to that obtained on the as-grown crystal is immediately apparent. Similar to the observation of the oxygen and copper XPS spectra obtained after oxygenating the crystal, we find a decrease in the relative intensity ratios of the peaks corresponding to (Cu-O-Cu-O)_*n*_ clusters and the (Al-Cu-O-Cu-O)_*n*_ clusters.

**Figure 9:**
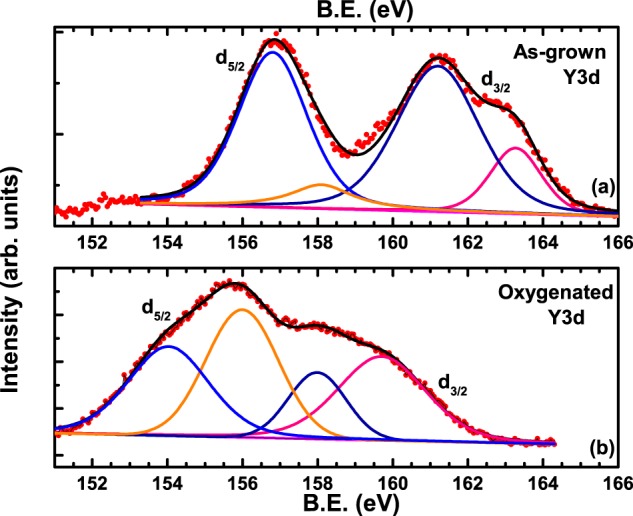
Core-level spectrum of Y-3d exhibiting 3d_5/2_ and 3d_3/2_ peaks in (as-grown) and (**b**) oxygenated crystal V_*bH*_.

Finally, we present the core-level spectrum of Y-3d in Fig. 9 which is characterised by a two-peak structure typical of 3-d core levels governed by spin-orbit coupling. The first feature at ~157 eV corresponds to the 3-d_5/2_ spin-orbit level while the second broad feature centred around 161 eV corresponds to the 3-d_3/2_ spin-orbit level. As usual, red filled circles correspond to data points while the superimposed black line is a fit to the data. The fit reveals four peaks, two centred around 3-d_5/2_ while the other two centred around 3-d_3/2_. The de-convoluted peaks are shown by blue and orange lines corresponding to 3-d_5/2_ while those belonging to 3-d_3/2_ are shown by dark blue and pink lines. Similar to previous observations, both the higher binding energy peaks (orange and pink lines) may correspond to (Al-Cu-O-Cu-..)_*n*_ clusters while the lower binding energy peaks (blue and dark blue lines) correspond to oxygen deficient clusters of the type (Cu-..-Cu-O)_*n*_ in the main lattice.

According to our proposition, the relative intensity of the peaks corresponding to (Al-Cu-O-Cu-..)_*n*_ clusters and (Cu-..-Cu-O)_*n*_ clusters should decrease after oxygenation due to the filling up of the oxygen deficient sites. This is exactly what is observed in the spectra of the oxygenated crystal shown in Fig. 9(b). The intensity of both the orange line as well as the pink line increases considerably after oxygenation. It was also found that the intensity ratios of the deconvoluted peaks (orange with respect to pink and light blue with respect to dark blue) occur roughly in the expected ratio of 3:2 corresponding to the multiplicity of the spin-orbit split features. The B.E. at which the main peaks of the 3-d_5/2_ and 3-d_3/2_ spin-orbit levels occur (orange and pink peaks respectively is around 156 eV and 159.5 eV respectively, similar to other reports^32,40,43^, in the parent YBCO.

## Conclusions

To conclude, systematic investigations of crystal structure and superconducting properties of Al doped YBCO, namely, YBa_2_Cu_3−*x*_Al_*x*_O_6+*δ*_ superconductor was done and the properties compared in the as-grown state *vis a vis* the oxygenated state. In contrast to previously reported works, orthorhombic crystal structures, but with very near values of lattice constants “a” and “b” were obtained in both as-grown as well as oxygenated single crystals. An oxygen vacancy cluster distribution model describing the presence of two different type of clusters of (Cu-O-Cu-..)_*n*_ and (Cu-O-Al-..-Cu)_*n*_ in the a-b plane of Al-YBCO crystals is proposed to explain the width in superconducting transition temperatures as well as differences in the magnetisation hysteresis loops of as-grown and oxygenated crystals of Al-YBCO. An experimental evidence of the proposed model is presented via X-ray photoelectron spectroscopy measurements where the spectrum of each of the element Y, Ba, Cu and O are found to have a two peak structure, wherein, each peak corresponds to one type of cluster. The relative ratio of the two peaks corresponding to each cluster is found to decrease after oxygenation as compared to that of the as-grown ones, as a result of a change in the number and distribution of the two types of clusters.
